# 10.O. Workshop: Developmental and School-Based Factors Shaping Sexual and Gender Minority Youth Mental Health

**DOI:** 10.1093/eurpub/ckac129.674

**Published:** 2022-10-25

**Authors:** Organised by: EUPHA-SGMH, EUPHA-CAPH, Chair persons: Arjan van der Star (EUPHA-SGMH), Richard Ma (UK)

## Abstract

Research from across the globe has consistently shown that young sexual and gender minority individuals (e.g., those identifying as lesbian, gay, bisexual, and/or transgender [LGBT+]) are at a higher risk for depression, anxiety, and suicidal thoughts and behaviors when compared to heterosexual youth, including during early childhood and later adolescence. A sizeable body of research has attributed the elevated risk to increased exposure to specific social stressors related to navigating a stigmatized minority identity, including stressors such as victimization and other interpersonal and social difficulties in, for example, the school environment. Yet, relatively less is known about the early developmental timing of such disparities in childhood and how LGBT+ youth navigate school climates. As a sensitive developmental period, childhood and adolescence may be a particularly challenging time for sexual and gender minority youth to navigate cisnormative and heteronormative school contexts. Exposure to oppressive norms, particularly in school environments, has only recently become the subject of research. Additionally, research has been limited on how supportive school climates may be protective but stigmatizing school environments may drive LGBT+ trajectories towards suicidal behaviors and shape how they may navigate self-disclosure of cooccurring identities and mental health status in school settings, particularly when such stigmas may prevalently intersect. This workshop aims to further explore these novel aspects around developmental and school-based risk and protective factors shaping the mental health of sexual and gender minority children and adolescents. This workshop includes five empirical presentations that span from examining the developmental timing of mental health disparities, the role school-based experiences play in shaping and driving these disparities, to how sexual and gender minority youth may navigate their school context and how supportive climates may be protective for mental health. First, Arjan van der Star will present longitudinal evidence on how sexual identity formation precedes the onset of sexual orientation-based mental health disparities and the role that peer difficulties play in driving these as early as pre-teen years. Next, Niolyne Jasmin Bomolo will present findings from a qualitative study that unravel how school-based experiences shape individual trajectories toward suicidal attempts among LGBT+ adolescents. Third, Wouter Kiekens will explore how normative cultures in school environments may drive sexual attraction-based mental health disparities among a large sample of adolescents. Fourth, Lourdes Cantarero Arévalo will present findings on how LGBT+ adolescents living with mental conditions navigate self-disclosure in school environments. Finally, Sandra Sevic will present results on how supportive school environments may be protective for gender minority mental health.

**Key messages:**

• Negative school-based experiences put sexual and gender minority youth at elevated risk for adverse mental health as early as middle childhood.

• Intersecting stigmas around minority identities and mental health problems may further complicate how sexual and gender minority children and adolescents navigate their school environments.

